# Alleviation of skin inflammation after Lin^−^ cell transplantation correlates with their differentiation into myeloid-derived suppressor cells

**DOI:** 10.1038/srep14663

**Published:** 2015-10-06

**Authors:** Su Jeong Ryu, Ji-Min Ju, Woojin Kim, Min Bum Kim, Kuen Hee Oh, Dong Sup Lee, Hakmo Lee, Ju Eun Oh, Kyong Soo Park, Eun Young Choi

**Affiliations:** 1 Department of Biomedical Sciences, Seoul National University College of Medicine, Seoul, Korea; 2 Biomedical Research Institute, Seoul National University Hospital, Seoul, Korea; 3 Department of Molecular Medicine and Biopharmaceutical Sciences, College of Medicine or College of Pharmacy, Seoul National University, Seoul, Korea; 4 Department of Internal Medicine, Seoul National University College of Medicine, Seoul, Republic of Korea

## Abstract

To understand the cellular mechanism underlying the therapeutic effects exerted by hematopoietic stem cell transplantation in the repair of tissue damage, we investigated the *in vivo* dynamics of bone marrow (BM) lineage-negative (Lin^−^) cells transplanted into mice with hyper sensitivity dermatitis. Longitudinal *in vivo* imaging and flow cytometry analyses revealed that Lin^−^ cells home directly to inflamed skin within 6 h, where they undergo extensive expansion with the peak on day 14 post-transplantation, and preferential differentiation into CD11b^+^Ly6G^int^Ly6C^+^ cells by day 7. Cells with phenotypic profiles of neutrophils, macrophages, and DCs appeared in inflamed skin on day 14. Progenies of transplanted Lin^−^ cells showed similar kinetics of expansion and myeloid differentiation in BM. However, differentiation into CD11b^+^Ly6G^int^Ly6C^+^ cells in the inflamed skin on day 7 was more skewed toward CD115^+^ cells (≥60%) with immune suppressive function and higher expression levels of iNOS, arginase, and IL-10, compared with those in the BM. Transplantation of Lin^−^ cells reduced the levels of *Cd3* transcript and CD4^+^/CD8^+^ cells in inflamed skin. These results demonstrate differentiation of transplanted Lin^−^ cells into myeloid-derived suppressor cells in inflamed skin to be the basis of the alleviation of skin inflammation after Lin^−^ cell transplantation.

Bone marrow (BM)-derived hematopoietic stem cells (HSCs) are recognized as self-renewing pluripotent cells capable of differentiating into a wide range of blood and immune cells. Recently, however, an alternative role of HSCs in the repair of parenchymal tissue inflammation has received much attention. Following peripheral tissue injury, endogenous HSCs are activated and mobilized from the BM, migrate to the site of inflammation, and facilitate tissue repair and wound healing^1^,^2^. Similar effects were reported for exogenously implanted HSCs, which homed to the site of damage and contributed to tissue repair, suggesting their potential for use in regenerative medicine^1^,^2^,^3^. However, despite these well-accepted effects of stem cell-based therapies, the underlying cellular mechanism has not been elucidated completely.

Migration to peripheral damaged sites and the pluripotent differentiation capacity of HSCs are the two major axes of their therapeutic potential. A growing number of molecular signals have been implicated in HSC migration. Multiple chemokines and proinflammatory cytokines (IL-1, IFN-α, IFN-β, TNF-α, and GM-CSF) produced at the site of inflammation were found to induce HSC-mobilization and tissue recruitment^3^,^4^,^5^. Chemokine receptors, such as CXCR4 and CCR2, along with adhesion molecules expressed on HSCs mediate their homing to the BM, and are considered important regulators of tissue recruitment^6^,^7^,^8^,^9^. Other than these molecular studies, the detailed cellular dynamics of exogenous HSCs, including distribution/migration behavior in the recipients, have not been investigated extensively due to the lack of tools to properly analyze the rare infused cells in the recipients. In terms of differentiation, HSCs were shown to differentiate into activated CD11b^hi^F4/80^lo^ macrophages upon reaching the site of inflammation in a drug-induced liver injury model^6^, indicating that the differentiation into these cells underlies a protective role for mobilized HSCs. Alternatively, in stroke, chronic heart disease, and hind limb ischemic models, HSCs were found to activate angiogenesis, which facilitated damage repair^10^,^11^,^12^. Otherwise, they differentiated into non-hematopoietic cells, contributing to the repair of skeletal and cardiac muscles, as well as skin injuries^13^,^14^,^15^. However, the underlying mechanism linking these various roles is unknown. Therefore, we conceived that longitudinal tracing of the differentiation of exogenous HSCs, in the context of *in vivo* dynamics including their homing/distribution and proliferation, would be essential for understanding how administration of exogenous HSCs provides regenerative benefits in parenchymal tissue repair.

To this end, we adopted various approaches to trace *in vivo* the fate of HSCs administrated exogenously. Bioluminescence imaging (BLI) analysis, which enables noninvasive *in vivo* cell monitoring^16^,^17^, was used to track luciferase-transgenic stem cells *in vivo* for longitudinal detection of the distribution, proliferation, and persistence of stem cells in recipients with parenchymal tissue damage, and flow cytometric analysis was used to evaluate concurrent differentiation of stem cells on a single-cell basis. We exploited the advantage of the enhanced luciferase sensitivity displayed in a recently developed luciferase transgenic mouse, which was successfully used for tracing immune cells *in vivo*^18^,^19^, and evaluated CD45.1 congenic marker expression in hematopoietic cells from CD45.1^+^ mice using these mice as stem cell donors (sources) in BLI and flow cytometric analyses, respectively. As an animal model for tissue inflammation, we used a model of allergic contact dermatitis elicited experimentally by local skin treatment of the chemical 2,4-dinitrocholorobenzene (DNCB). The disease involves T cell activation, along with infiltration of neutrophils and monocytic cells to the inflammatory lesion^20^,^21^. TNF-α, IL-1, and IFN-γ are produced at high levels, while IL-10 is detected at low levels, during the course of the disease^5^,^20^. This model allowed us to evaluate the anti-inflammatory effect of stem cell transplantation using the naked eye and to visualize migration of the luciferase-expressing stem cells to the inflamed skin more efficiently, as detection of luminescence signals is relatively straightforward if the signals emanate from locations in the vicinity of the body exterior, rather than the interior. Additionally, the well-established immunological pathogenesis of the disease facilitated investigation of the mechanism underlying the anti-inflammatory effect of the stem cells in an immunological context.

In this study, we performed longitudinal BLI and flow cytometric analyses of exogenously administered HSCs, using lineage-negative (Lin^−^) cells in BM, which do not express lymphoid or myeloid lineage markers. These approaches revealed that the therapeutic effects generated by transplanted Lin^−^ cells depend on their targeted migration to sites of inflammation and subsequent expansion. Intriguingly, we found that at the inflamed site, these cells differentiated into myeloid-derived suppressor cells (MDSCs), which restrain the immune response in various settings, including cancer, inflammation and infection^22^. Here, providing detailed information regarding the *in vivo* fate of exogenously administered HSCs, we demonstrate that expansion and concurrent differentiation into MDSCs *in situ* at the site of local inflammation are correlated with the therapeutic effect of HSC transplantation.

## Results

### Transplantation of BM lineage-negative cells alleviates skin inflammation in mice with DNCB-induced contact hypersensitivity dermatitis

**Figure 1:**
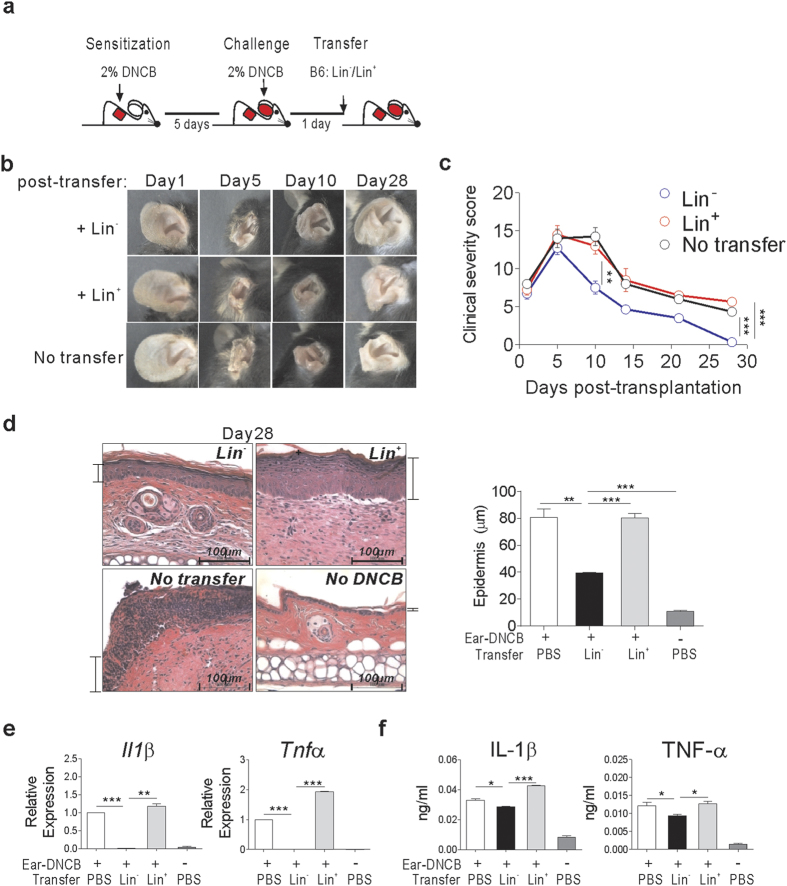
Transplantation of BM Lin^−^ cells enhances healing and skin regeneration in dermatitis mice. (**a**) Schematic overview of DNCB treatment and Lin^−^ cell transplantation. Contact hypersensitivity dermatitis was induced in B6 mice by applying 2% DNCB solution to their shaved back, followed by a secondary challenge to the right ear 5 days later. One day after the secondary challenge, Lin^−^ or Lin^+^ cells (5 × 10^6^) isolated from the BM of normal B6 mice were injected i.v. into dermatitis mice. (**b**,**c**) The severity of ear skin inflammation after challenge with DNCB at several time points after transplantation (**b**), and the longitudinal clinical scores of these mice (**c**). Longitudinal photos of same mouse are presented in (**b**). Clinical severity scores in (**c**) were graded on a scale of 0 to 4, as described in Materials and Methods, periodically at 1 to 28 days after transplantation. (**d**) Histological examination of the inflamed ear skin of dermatitis mice receiving Lin^−^ or Lin^+^ cell-transplantations, along with negative controls that received PBS (No transfer). Representative H&E stained images of mice harvested 28 days after transplantation are shown (Scale bar, 100 μm); ear skin from control mice without DNCB treatment (no DNCB) was examined in parallel. Indications of epidermal layer are added to each image, with the depths of each experimental group are plotted. (**e**,**f**) Expression of IL-1β and TNF-α in the skin and blood of mice. (**e**) Relative expression of *Il1β* and *Tnfα* were analyzed by qRT-PCR using RNA harvested from the skin tissue on day 14 post-transplantation. Delta-delta Ct values were normalized to those obtained from amplification of β-actin and were expressed as fold changes compared to gene profiles of the DNCB with PBS-transfer sample. (**f**) Levels of IL-1β and TNF-α in the serum harvested from mice 14 days post-transplantation were measured by ELISA. Data (**b-f**) are representative of more than three independent experiments (n = 4 mice/group/experiment). Data (**b-f**) are presented as means ±SEM. *P* values were determined using two-tailed unpaired Student’s *t*-tests; *P < 0.05, **P < 0.01, ***P < 0.001.

To determine whether HSC transplantation contributes to skin regeneration through alleviating the inflammation, lineage marker-negative (Lin^−^) cells isolated from BM were administered intravenously (i.v.) into mice with dermatitis. We used Lin^−^ cells as HSCs, since Lin^−^ cells are not only capable of multi-potent differentiation but are also frequently used as primitive HSCs in regenerative medicine^23^. Additionally, these cells have been shown to exhibit superior healing effects compared to highly purified HSCs in a full-thickness wound model^15^,^24^. 2% DNCB was the marginal low dose used to induce inflammation with apparent clinical scores. Sensitization of the back skin of B6 mice with DNCB, followed by a secondary application to the right ear 5 days later, induced severe inflammation at the ear (Fig. 1a), as reported previously^21^. The inflammation peaked between 5 and 7 days after the secondary DNCB application with edema, serious skin necrosis, and evident loss of ear shape, and required more than 30 days for full recovery (Fig. 1b–d). Transplantation of Lin^−^ cells 1 day after the ear-challenge resulted in improved tissue regeneration, and almost complete restoration of ear shape by day 28 (Fig. 1b–d). Compared with the ears of control mice infused with Lin^+^ cells or PBS (no transfer), the ears of the Lin^−^ cell recipients showed reduced leukocyte infiltration and thickening of the epidermal layer on day 28 post-transplantation, but with incomplete tissue regeneration and some residual skin inflammation under microscopic examination (Fig. 1d). Transcript levels of *Il-1β* and *Tnf-α*, inflammatory cytokines known to be involved in DNCB-induced dermatitis^20^,^21^, were significantly lower in the inflamed ear skin tissues of Lin^−^ cell-transplanted mice than in controls, when measured on day 14 (Fig. 1e). Also the serum cytokine levels were lower in the Lin^−^ cell-transplanted mice than in those of control mice, indicating relatively low inflammatory systemic status of the former (Fig. 1f). Together, these data indicated a marked therapeutic effect of Lin^−^ cell transplantation on skin inflammation in DNCB-induced dermatitis mice.

### Longitudinal *in vivo* dynamics of transplanted Lin^−^ cells in dermatitis mice

**Figure 2:**
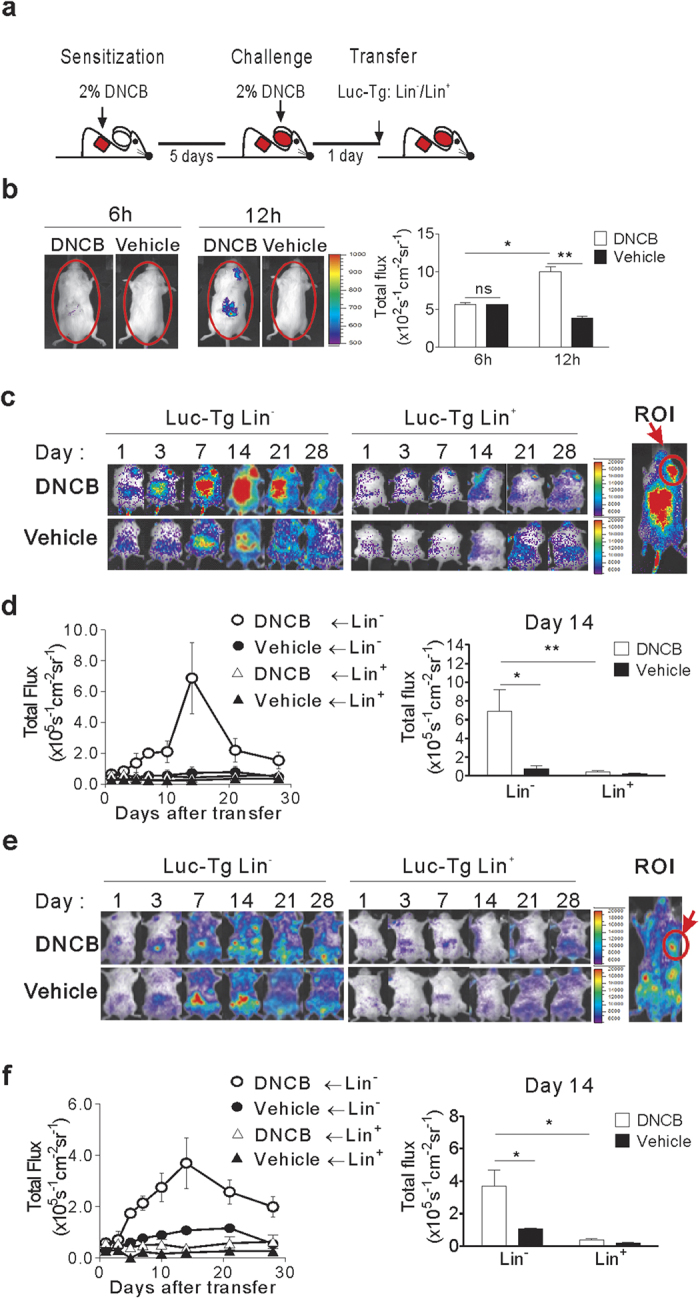
*In vivo* dynamics of transplanted Lin^−^ cells in dermatitis mice. (**a**) Schematic overview of DNCB-treatment and Lin^−^ or Lin^+^ cell transplantation. Lin^−^ and Lin^+^ cells were isolated from Luc-Tg mice. (**b**) Bioluminescence images were taken 6 and 12 h post-transplantation of Luc-Tg cells (5 × 10^6^). Dorsal images are shown. Total photon counts were generated using the entire dorsal area of the mouse as the region of interest (ROI). (**c**,**d**) Longitudinal dorsal bioluminescence images. (**c**) Representative dorsal images of dermatitis or control mice transplanted with Luc-Tg Lin^−^ or Lin^+^ cells (5 × 10^5^) were taken on days 1, 3, 7, 14, 21, and 28 post-transplantation. The inflamed right ear was designated as the ROI. (**d**) Photon flux values emanating from the right ear of the mice were plotted longitudinally. Peak signal values were compared between experimental groups on day 14 post-transplantation. (**e**,**f**) Longitudinal ventral bioluminescence images. Ventral images were processed as above (**c**,**d**) after designation of the upper tibia as the ROI. Data shown (**b**–**f**) are representative of more than three independent experiments (n = 4 mice/group/experiment). Bar graphs in (**b**,**d**,**f**) are presented as means ±SEM. *P* values were determined using two-tailed unpaired Student’s *t*-tests; ns, not significant, *P < 0.05, **P < 0.01.

Then, we traced the *in vivo* dynamics of exogenous Lin^−^ cells in the DNCB-induced dermatitis mice via BLI analysis. DNCB was treated to B6-Albino mice and these mice were transplanted with Lin^−^ cells isolated from the BM of luciferase transgenic mice (B6.Luc-Tg; Luc-Tg mice), which express the enhanced firefly luciferase gene under the control of the actin promoter^18^. Then, images of the luminescent signals emitted by the transplanted cells were obtained periodically (Fig. 2a). Mice receiving a primary DNCB-sensitization on the back only, followed by a secondary vehicle-only challenge, were used as a control (vehicle control) without ear inflammation.

Transplantation of Lin^−^ cells isolated from Luc-Tg mice alleviated skin inflammation in albino dermatitis mice as well (Fig. S1, Supplementary). In dorsal images of the dermatitis mice, luminescence signals were detected in the back and inflamed right ear within 12 h post-transplantation, with faint signals evident within 6 h (Fig. 2b). Longitudinal data demonstrated that luminescence signals were consistently strongest at the sites of inflammation: the skin of the back and right ear (Fig. 2c). When the intensities of the signals emanating from the inflamed right ear, designated as a region of interest (ROI), were measured and plotted, it was evident that the signal increased consistently over the first 14 days; this implied a significant increase in the number of exogenous cells at the inflamed right ear, which waned significantly after the peak on day 14 post-transplantation (Fig. 2d). In dorsal images of vehicle-control mice receiving primary DNCB treatment at the back only, luminescence was detected only at the later time points and only in the back skin, but with significantly lower intensity compared with the signals detected in the back of mice with dermatitis (Fig. 2b,c). Since the sensitized back skin looked more inflamed in the mice with DNCB-ear challenge than in controls, the difference in signal intensities detected at the back skin between the two groups of mice was considered to reflect the different degrees of inflammation at the primary sites.

In the ventral images, Lin^−^ cell luminescence was detected at multiple BM sites in both dermatitis and vehicle-treated controls (Fig. 2e). This localization is thought to reflect their capacity as stem cells for selective homing to the BM, regardless of other conditions. Signal intensities from a tibia BM site in the left foreleg were measured longitudinally, which was chosen as a ROI due to its physical distance from the abdomen where false positive luminescence signals were often generated as a result of intraperitoneal (i.p.) injection of the substrate luciferin even in the absence of Luc-Tg cells in the abdomen. Plotting of the signal intensities emanating from ROI demonstrated drastic signal increases in the BM at this site only in dermatitis mice (Fig. 2f). The kinetics of signal changes in the BM sites of dermatitis mice were similar to that detected in the inflamed ears in dorsal images of the same dermatitis mice (Fig. 2d).

In a parallel experiment in which dermatitis or vehicle-treated control mice received Lin^+^ cells isolated from Luc-Tg mice (Fig. 2c–f), luminescent Lin^+^ cells were detected in the inflamed ear and back skin, as well as BM, but the intensities were significantly lower than the signals observed from Lin^−^ cells. Lin^−^ and Lin^+^ cells purified from the BM of Luc-Tg mice express similar levels of the luciferase transgene, emitting similar levels of luminescence before transplantation (average of 0.38 and 0.49 s/cm^2^/sr per cell, respectively), and luciferin substrate diffuses fast and evenly to almost every tissues^18^ (Fig. S2, Supplementary). Therefore, any change in signal intensity was regarded as a change in the number of corresponding cells.

Taken together, the BLI data demonstrated that transplanted cells were recruited to inflamed skin and BM of dermatitis mice, regardless of whether Lin^−^ or Lin^+^ cells were used, and that long lasting and drastic increase of luminescence signals were specific to the progenies of Lin^−^ cells. Additionally, they demonstrate increases in signal intensities with similar kinetics both in the inflamed skin and BM, as well as a positive correlation between the degree of signal increase and inflammation in the recipients.

### Homing and expansion of transplanted Lin^−^ cells in the BM and inflamed skin of dermatitis mice

**Figure 3:**
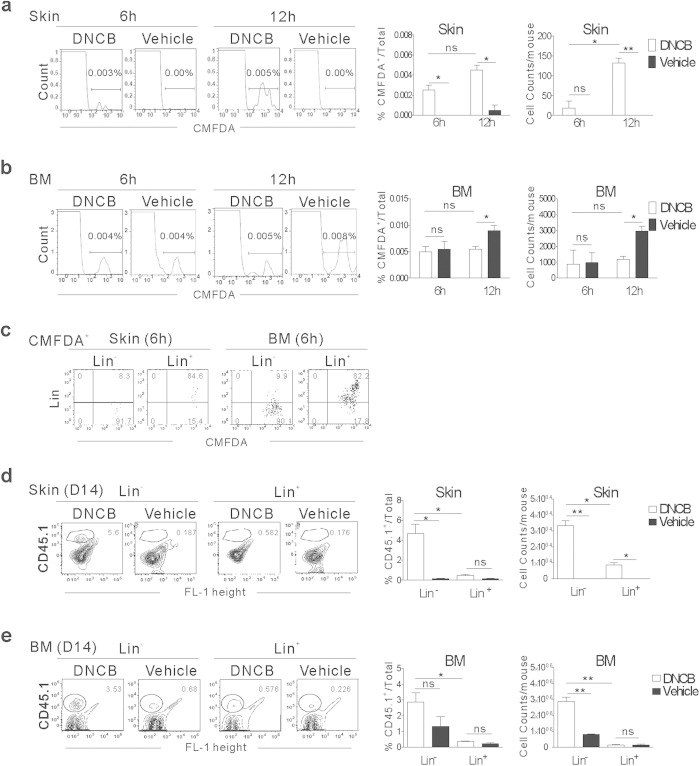
Early homing and expansion of Lin^−^ cells at the site of inflammation and BM. (**a–c**) Flow cytometric analysis of CMFDA^+^ cells in inflamed skin and BM at 6 and 12 h after transplantation of CMFDA^+^CD45.1^+^Lin^−^ cells. Dermatitis or vehicle-control CD45.2^+^ B6 mice were transplanted with CMFDA-labeled Lin^−^ cells or Lin^+^ cells (1 × 10^6^), and analyzed for the presence of CMFDA^+^ cells in the inflamed skin and BM at 6 and 12 h after the transplantation by flow cytometry; representative flow cytometric data are shown. The percentage of CMFDA^+^ cells in the leukocytes present in skin (**a**) or BM cells (**b**), and the numbers of CMFDA^+^ cells in the skin and BM per mouse are shown. (**c**) Flow cytometric analysis of lineage marker expression in CMFDA^+^ cells in inflamed skin and BM at 6 h post-transplantation of CMFDA-labelled CD45.1^+^Lin^−^ or Lin^+^ cells. Representative data are shown. (**d**,**e**) Flow cytometric analysis of CD45.1^+^ cells in inflamed skin (**d**) and BM (**e**) 14 days post-transplantation of CD45.1^+^Lin^−^ or Lin^+^ cells; representative data are shown. Data were processed as described in (**a**,**b).** Data shown (**a**-**e**) are representative of more than three independent experiments (n = 3 mice/group/experiment). Data (**a**,**b** and **d,e**) are presented as means ±SEM. *P* values were determined using two-tailed unpaired Student’s *t*-tests; ns, not significant, *P < 0.05, **P < 0.01.

The similar expansion kinetics of Lin^−^ progenies in the inflamed skin and BM suggested that Lin^−^ cells home to the skin and BM almost immediately after transplantation, although the signals from BM were rarely detected in the ventral BLI data at early time points (data not shown). To examine this on a single-cell basis, we performed flow cytometric analysis of cells infiltrating the inflamed skin and BM of CD45.2^+^ B6 dermatitis mice following transplantation of 5-chloromethylfluorescein diacetate (CMFDA)-labeled CD45.1^+^Lin^−^ cells. CMFDA-positive (CMFDA^+^) cells were detected among the cells infiltrating the inflamed skin and in the BM of the upper tibia at 6 and 12 h post-transplantation (Fig. 3a,b), and even after the recipient mice were perfused with PBS prior to the analysis at 12 hr, to exclude the cells in blood (Fig. S3, Supplementary). The CMFDA^+^ cells in the inflamed skin were of lineage marker-negative status at 6 h (Fig. 3c). Taken together, these data indicate early or immediate homing of Lin^−^ cells, following upon transplantation into inflamed sites as well as the BM.

The absolute numbers of transplanted (CMFDA^+^ or CD45.1^+^) cells in the inflamed skin of dermatitis mice increased between 6 and 12 h (Fig. 3a), up to an average of 3.2 × 10^4^ cells (4.4% of leukocytes in the inflamed skin) on day 14 post-transplantation (Fig. 3d). However, these absolute numbers in the inflamed skin were lower than those in the BM of the dermatitis mice even at 6 h (Fig. 3b), signifying that homing to the BM was a major event following transplantation, even with a subpopulation of these cells preferentially homing to inflamed skin. The percentage and absolute number of Lin^−^ cells in the BM were similar between the dermatitis mice and vehicle-control mice at 6 h post-transplantation, but eventually increased significantly (up to 2.8% of BM cells) in the dermatitis mice on day 14 post-transplantation (Fig. 3e), after a transient stagnation between 6 hr and 12 hr (Fig. 3b, Fig. S3, Supplementary). This transient stagnation of Lin^−^ cells in BM of the dermatitis recipients may be due to the presence of continued recruitment of Lin^−^ cells to inflamed sites by the 12 hr time point, while such recruitment rarely occurring in the control mice. Such substantial increase in the CD45.1^+^Lin^−^ progenies in the inflamed skin and BM observed only in dermatitis mice, compared with control mice, is consistent with the BLI data (Fig. 2).

CD45.1^+^Lin^+^ cells were also recruited to the site of inflammation and to the BM of dermatitis mice (Fig. 3c); however, their proportions and absolute numbers were significantly lower in both locations than those of CD45.1^+^Lin^−^ progenies on day 14 (Fig. 3d,e).

Taken altogether, these data demonstrate considerable early homing of transplanted Lin^−^ cells to inflamed skin as well as to the BM, and subsequent expansion of the progenies at both sites in the dermatitis mice, confirming the BLI studies.

### *In situ* proliferation of progenies of transplanted Lin^−^ cells in the inflamed skin

**Figure 4:**
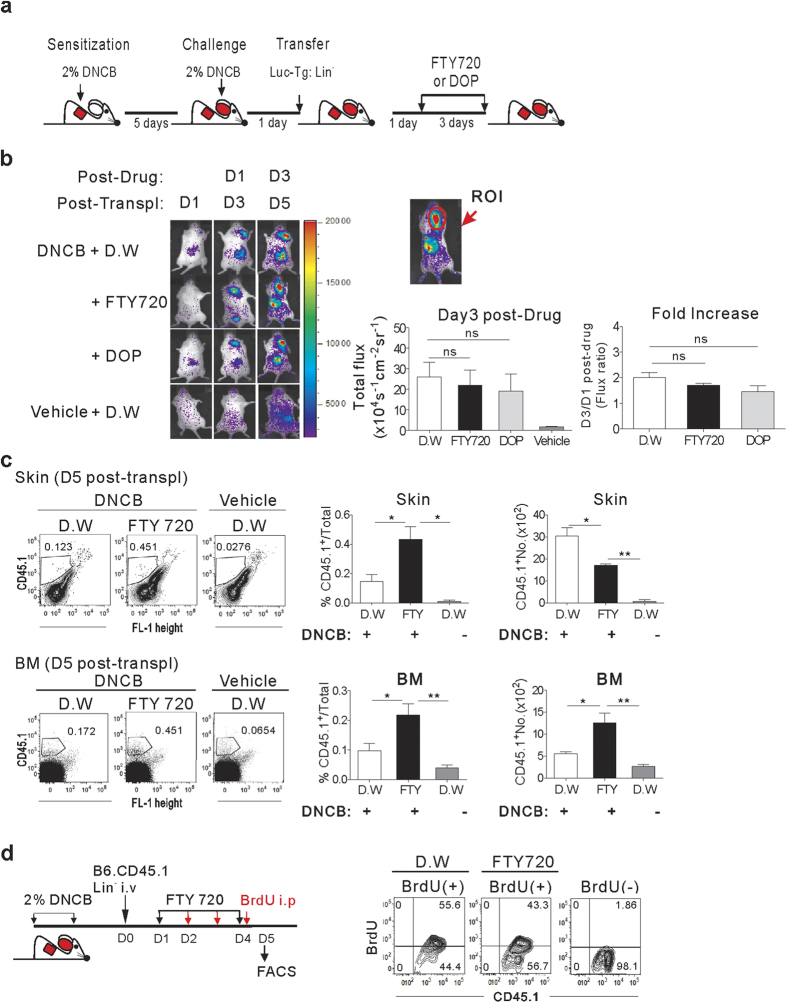
*In situ* proliferation of Lin^−^ cells at the site of inflammation. (**a**) Schematic overview of DNCB treatment, Lin^−^ cell transplantation, and BM egress inhibition. FTY-720 or DOP was added to the drinking water for 3 days, beginning 1 day after transplantation of Lin^−^ cells. (**b**) Dorsal bioluminescence images of transplanted (5 × 10^5^ of Luc-Tg Lin^−^ cells) and drug-treated mice. Total photon flux values from the right ear were measured, and fold increases between days 1 and 3 post-drug treatment are shown. (**c**) Flow cytometric analyses of CD45.1^+^Lin^−^ cells in the skin and BM of the transplanted (1 × 10^6^ of Lin^−^ cells from CD45.1^+^ mice) and drug-treated dermatitis mice on day 5 post-transplantation; representative data are shown. The percentage of CD45.1^+^ cells in the leukocytes present in skin or BM cells and the numbers of CD45.1^+^ cells in the skin and BM per mouse are plotted. (**d**) DNCB-treated, CD45.1^+^Lin^−^ cell-transplanted, and FTY-720-treated mice were injected with 1 mg of BrdU daily for 3 days. FTY-720 was added to drinking water continuously until day 4 starting from day 1 post-transplantation. Leukocytes were isolated from the skin and stained with anti-CD45.1-APC and anti-BrdU-FITC antibodies. Representative flow cytometric data shown are gated for CD45.1^+^ cells. Data shown (**b**–**d**) are representative of two independent experiments (n = 5 mice/group/experiment). Data (**b**,**c**) are presented as means ±SEM. *P* values were determined using two-tailed unpaired Student’s *t*-tests; ns, not significant, *P < 0.05, **P < 0.01.

We wondered whether the signal increases observed at the site of inflammation 14 days post-transplantation were the result of *in situ* proliferation of Luc-Tg Lin^−^ progenies already present in the inflamed skin, or constant influx of cells originally homing to the BM. To resolve this, we treated dermatitis mice with the cell egress blockers FTY720 or 4-deoxypyridoxine (DOP), which retain cells in BM for 24 h^25^,^26^, continuously from one day after transplantation of Luc-Tg Lin^−^ cells, and monitored the increase in the luminescence signal in the inflamed ear (Fig. 4a). Continued exposure to cell-egress blockers over 3 days failed to inhibit signal increases in the inflamed skin (Fig. 4b), with an average 1.7- or 1.5-fold increase in signal intensities detected in the inflamed ear skin during the drug treatment period (day 3/day 1 post-FTY720 or DOP treatment, respectively). This indicated that *in situ* expansion of Lin^−^ progenies at the site of inflammation contributed to the signal increase in the absence of a cellular influx from the BM. However, these fold-change values were slightly lower, albeit not significantly so, compared to the values (2.0-fold on average) in the DW-treated group. Therefore, it is possible that cellular influx from the BM also contributes to the increase in Lin^−^ progenies in the inflamed skin in the absence of such a BM-egress blockade.

Flow cytometric analyses of mice treated with the cell egress blocker FTY-720 revealed a marked increase in the proportion of CD45.1^+^ cells in the inflamed skin of dermatitis mice, indicative of *in situ* expansion of these cells in the absence of cellular egress from BM. However, their absolute number in the inflamed skin was lower in the presence of cell egress blockers relative to DW-fed controls (Fig. 4c). Therefore, in addition to their proliferation at inflamed sites, influx of cells from the BM also accounted for the increase in the CD45.1^+^ Lin^−^ progenies in the inflamed skin. FTY720 treatment enhanced both the percentages and numbers of CD45.1^+^ cells in the BM, verifying the blockade of cell egress from BM during drug treatment. The presence of *in situ* proliferation of Lin^−^ progenies was further confirmed by the presence of BrdU-positive cells among the CD45.1^+^ cells in inflamed skin, following BrdU administration after initiation of FTY-720 treatment (Fig. 4d).

### Transplanted Lin^−^ cells differentiate into myeloid and granulocytic cells

**Figure 5:**
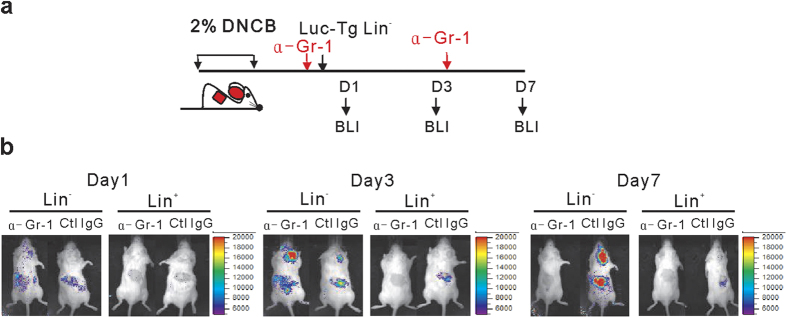
Differentiation of Lin^−^ cells into myeloid and granulocytic cells at the site of inflammation. (**a**) Schematic overview of treatment with anti-Gr-1 depleting antibody and transplantation of Lin^−^ or Lin^+^ cells (5 × 10^5^). Dermatitis mice were injected i.p. with an anti-Gr-1 antibody prior to and 3 days after transplantation with Luc-Tg Lin^−^ cells; rat-IgG was treated as a control. (**b**) BLI images were collected on days 1, 3, and 7 post-transplantation. Images shown are representative of two independent experiments (n = 3 mice/group/experiment).

Having established the source of cells expanded at the site of inflammation, we next investigated differentiation of Lin^−^ progenies at the site of inflammation, as these processes were presumed to play a role in their therapeutic effects. To determine the lineage of the differentiating cells, we selectively depleted each cell lineage by treating the recipients with antibodies against lineage markers (Gr-1, CD4, CD8, or B220) prior to and 3 days after transplantation of Luc-Tg Lin^−^ cells (Fig. 5a). Then, differentiation into a specific lineage was determined by specific signal abrogation in the recipients with the corresponding depletion treatment in BLI analyses. Treatment with a Gr-1-depleting antibody abrogated the luminescence signals at the site of inflammation when the recipients of Luc-Tg Lin^−^ cells were analyzed on day 7, but not on days 1 or 3 post-transplantation (Fig. 5b), as did clodronate-treatment which depletes macrophage/monocytes^27^ (data now shown). This indicated depletion of Gr-1-expressing cells originated from transplanted Luc-Tg Lin^−^ cells in the inflamed skin on day 7. Prior flow cytometric analysis had verified depletion of Gr-1^hi^ granulocytes in the inflamed skin of dermatitis mice with one time treatment of anti-Gr-1 antibody (data not shown). Other treatments including those targeting CD8, CD4, and B220 had no effect on signal detection (data not shown). These results indicated that, despite their multi-lineage potency, Lin^−^ cells preferentially differentiated into myeloid and granulocytic lineage cells within the first 7 days, in consistent with the promoted myeloid/granulocytic differentiation in the presence of inflammation, relative to steady-state hematopoiesis^5^,^28^.

### Myeloid/granulocytic differentiation of transplanted Lin^−^ cells occurs similarly in inflamed skin and BM

**Figure 6:**
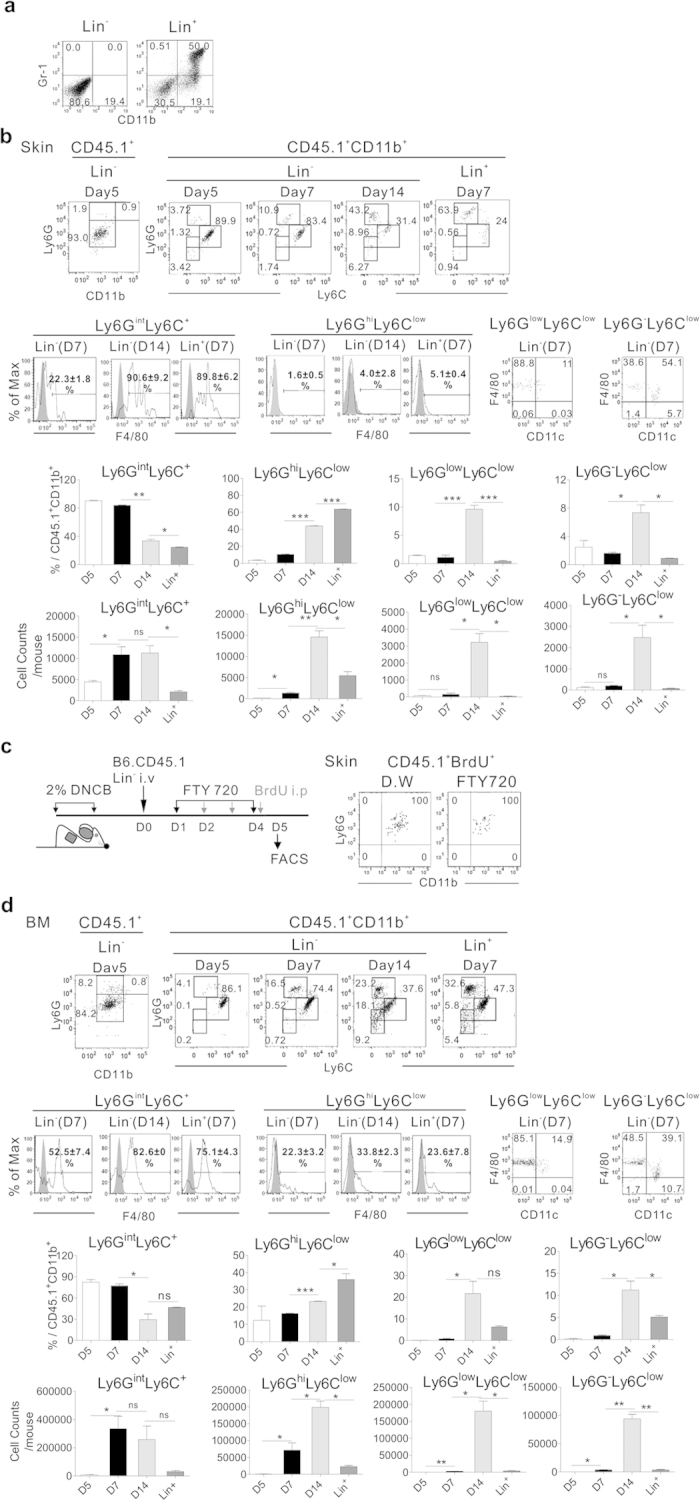
Differentiation of CD45.1^+^Lin^−^ cells into myeloid and granulocytic lineages in the inflamed skin and BM of the dermatitis mice. (**a**) Flow cytometric analysis of Lin^−^ or Lin^+^ cells purified for transplantation. (**b**) Flow cytometric analysis of CD45.1^+^ cells in the inflamed skin of dermatitis mice transplanted with CD45.1^+^Lin^−^ or Lin^+^ cells (1 × 10^6^) at various time points after transplantation. CD45.1^+^ cells isolated from the skin were analyzed for expression of CD11b, Ly6G, Ly6C, F4/80, and CD11c on the designated days post-transplantation. Representative data gated for CD45.1^+^ cells are shown. Percentage values of each population within the CD45.1^+^CD11b^+^ cells and absolute cell numbers are shown. (**c**) DNCB-treated, CD45.1^+^Lin^−^ cell-transplanted, and FTY-720-treated mice were injected with 1 mg BrdU daily for 3 days. FTY-720 was added to drinking water continuously until day 4 starting from day 1 post-transplantation. Leukocytes were isolated from the skin and stained with anti CD45.1-eFluor 450, anti-CD11b APC, anti-Ly6G PE and anti-BrdU-FITC antibodies. Representative data gated for CD45.1^+^BrdU^+^ cells are shown (**d**) Flow cytometric analysis of CD45.1^+^ cells in the BM. Data were analyzed as described above; representative data gated for CD45.1^+^ cells are shown. Data (**a**-**d**) are representative of three independent experiments (n = 5 mice/group/experiment). Data (**B,d**) are presented as means ±SEM. *P* values were determined using two-tailed unpaired Student’s *t*-tests; ns, not significant, *P < 0.05, **P < 0.01, ***P < 0.001.

To better characterize the myeloid lineage of Lin^−^ progenies, we used flow cytometry to examine CD45.1^+^ cells in the inflamed skin on days 5 and 7 post-transplantation of CD45.1^+^Lin^−^ cells when the ear inflammation was severe, and on day 14 when the skin inflammation subsided significantly (Fig. 1), supposing that healing effects of Lin^−^ cell transplantation would be exerted before inflammation begins to subside. Lin^−^ cells isolated from CD45.1^+^ donor mice were negative for CD11b and Gr-1 (Ly6G and Ly6C; Fig. 6a), as well as B220, CD3, Ter119 and CD11c expression (data not shown). However, when analyzed on day 5 post-transplantation, all of the CD45.1^+^ cells of donor origin were CD11b^+^ (Fig. 6b), consistent with a myeloid cell lineage. A large majority (≥80%) of CD11b^+^ cells exhibited a Ly6G^int^Ly6C^+^ phenotype on days 5 and 7 post-transplantation. However, on day 14, the proportions of Ly6G^int^Ly6C^+^ cells were reduced dramatically (33.6% on average), although their absolute numbers were not changed significantly, compared to those on day 7. Instead, Ly6G^hi^Ly6C^low^F4/80^−^ neutrophils were detected at larger proportions (43%), with their numbers being significantly increased, along with Ly6G^low^Ly6C^low^ (9.6%) and Ly6G^−^Ly6C^low^ (7.4%) cells, which were composed of F4/80^+^ macrophages and CD11c^+^ dendritic cells^22^,^29^.

CD11b^+^Ly6G^int^Ly6C^+^ was the phenotype of the BrdU-incorporating cells undergoing *in situ* proliferation at the inflamed skin site during the period of FTY-720 BM-egress blocker treatment (Fig. 6c), suggesting that proliferating cells *in situ* would differentiate into CD11b^+^Ly6G^int^Ly6C^+^ cells in the inflamed skin. A majority of the Ly6G^int^Ly6C^+^ cells on days 7 and 14 were positive for F4/80, but the surface F4/80 levels were higher on the day 14-cells, indicating that these cells are more matured status compared to day 7-cells. Taken together, these data show the predominance of the Ly6G^int^Ly6C^+^ cells with *in situ* proliferative potential over the first 7 days and the later appearance of other myeloid cells in the inflamed skin on day 14, and suggest that the CD11b^+^Ly6G^int^Ly6C^+^ cells might be the precursors of the late appearing matured myeloid cells.

Interestingly, compositions of CD45.1^+^Lin^−^ cells in the BM at the same time points post-transplantation were quite similar to those in inflamed skin, with the Ly6G^int^Ly6C^+^F4/80^+^ cells representing the major population among the CD45.1^+^ cells in BM, at days 5 and 7 post-transplantation (Fig. 6d).

In contrast to the dynamic changes in myeloid populations derived from Lin^−^ cells over time, progenies of transplanted Lin^+^ cells comprised Ly6G^hi^Ly6C^low^F4/80^−^ neutrophils (≥60%), along with smaller proportions of Ly6G^int^Ly6C^+^F4/80^+^ (24.5% on average) and Ly6G^−/low^Ly6C^low^ cells (≤10%) in the inflamed skin (Fig. 6a). Unlike Lin^−^ cells, the relative proportions of these cell populations remained stable throughout the analysis period, indicating a clear difference between Lin^−^ and Lin^+^ cells.

These data led to us conclude that transplanted Lin^−^ cells preferentially differentiated into cells of myeloid and granulocytic lineage in the inflamed skin as well as in the BM of dermatitis mice with similar kinetics. Moreover these results demonstrated that transplanted Lin^−^ cells differentiated mostly into CD11b^+^Ly6G^int^Ly6C^+^ cells with proliferative potential on days 5–7 post-transplantation, when the inflammation was most severe.

### Enhanced differentiation of Lin^−^ cells into CD11b^+^Ly6G^int^Ly6C^+^CD115^+^ MDSCs in the inflamed skin

**Figure 7:**
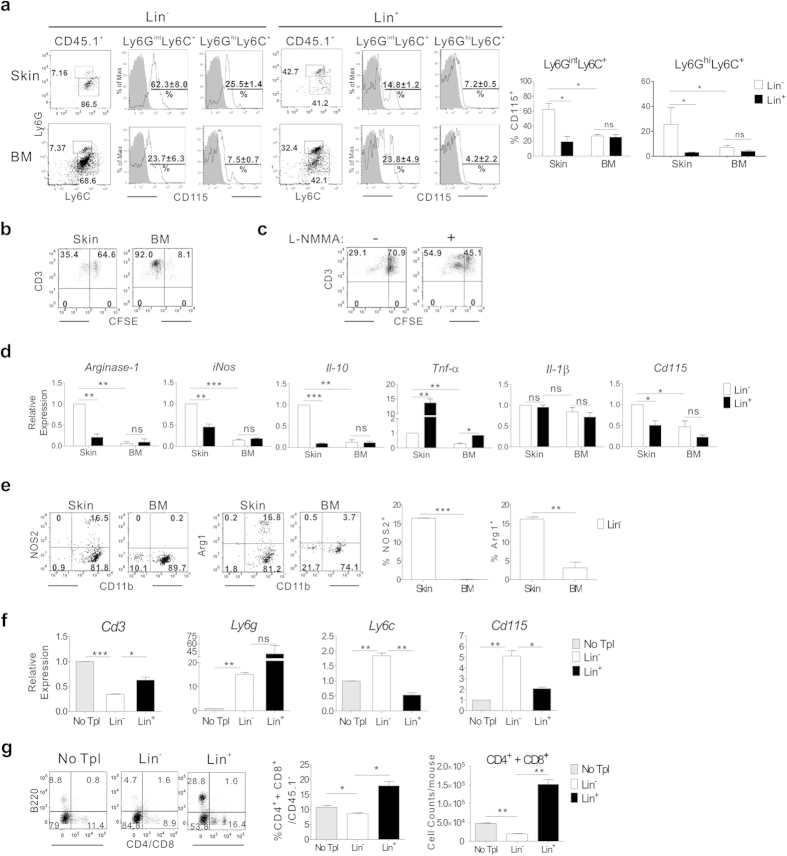
Differentiation of CD45.1^+^Lin^−^ cells into CD115^+^ MDSCs in inflamed skin. (**a**) Flow cytometric analysis of CD45.1^+^ cells in the inflamed skin and BM of recipients of CD45.1^+^Lin^−^ or Lin^+^ cells on day 7 post-transplantation. CD45.1^+^ cells were analyzed by flow cytometry and representative data are shown. Percentage values of CD115^+^ cells within the CD11b^+^Ly6G^int^Ly6C^+^ and CD11b^+^Ly6G^hi^Ly6C^+^ cell populations are plotted. (**b**,**c**) Immune suppression assays of CD45.1^+^ cells isolated from the inflamed skin or BM (**b**), or those from skin in the presence (+) or absence (−) of L-NMMA (1 mM). CFSE dilution by CFSE-labeled CD3^+^ T cells was analyzed via flow cytometry after stimulation in the presence sorted CD45.1^+^ cells. (**d**) qRT-PCR analysis of CD45.1^+^ cells isolated from the skin and BM. Relative levels of transcripts expressed by CD45.1^+^ cells in the BM and skin of mice transplanted with Lin^−^ cells and Lin^+^ cells were compared. Delta-delta Ct values were normalized to those obtained from amplification of β-actin and were expressed as fold changes compared with gene profiles of Lin^−^ cells in inflamed skin. (**e**) Flow cytometric analysis for expression of CD11b and NOS2 by CD45.1^+^ cells in the inflamed skin and BM of recipients of CD45.1^+^Lin^−^ cells on day 7. Representative flow cytometric data are shown, and the percentage values of NOS2^+^ or arginase-1^+^ cells within the CD45.1^+^ cell populations are plotted. (**f**) qRT-PCR analysis using RNA extracted from the inflamed skin of Lin^−^ or Lin^+^ cell recipients, along with negative control mice that received PBS (no Tpl; no transplantation). Relative transcript levels are shown. Normalized values are expressed as fold changes compared with gene profiles of the no transplantation sample. (**g**) Flow cytometric analysis of skin-infiltrating cells after staining with anti-CD4-PE, anti-CD8-PE, anti-B220-APC, anti-CD45.1-eFlour 450 antibodies on day 7 post-transplantation. Representative flow cytometric data are shown after CD45.1^−^ cell gating. Data shown (**a**–**g**) are representative of two independent experiments (n = 3–5 mice/group/experiment). Data (**a**,**d**–**g**) are presented as means ±SEM. *P* values were determined using two-tailed unpaired Student’s *t*-tests; ns, not significant, *P < 0.05, **P < 0.01, ***P < 0.001.

The CD11b^+^Ly6G^int^Ly6C^+^ phenotype with proliferative potential is consistent with their classification as MDSCs, which have been well characterized to have an immunosuppressive function in tumor environments^22^. Mouse MDSCs are known to express CD115^30^. Thus, we examined the expression of CD115 by the Ly6G^int^Ly6C^+^ cells of transplanted CD45.1^+^Lin^−^ cell origin in the inflamed skin and BM on day 7, after which disease scores decrease (Fig. 1c), implying a high probability that Lin^−^ progenies are exerting suppressive function about the time point. The proportion of CD115^+^ cells in the predominant Ly6G^int^Ly6C^+^ cell population was significantly elevated (62.4% on average) in the inflamed skin, with little decrease in the proportion of CD115^+^ cells among the minor Ly6G^hi^Ly6C^+^ cell population (24.45%; Fig. 7a). Unexpectedly, however, proportions of CD115^+^ cells were significantly lower (27% on average) among the Ly6G^int^Ly6C^+^ cells in the BM. These results show that, despite the similarity in the overall phenotypes of the Ly6G^int^Ly6C^+^ cells between the inflamed skin and BM, differentiation into CD115^+^ cells was preferential in the inflamed skin. As an additional control, Lin^+^ cells in inflamed skin comprised low proportions of CD115^+^ cells (19.3% on average).

Next, we evaluated the suppressive functions of the CD45.1^+^ cells present in the inflamed skin and BM on day 7 post-transplantation of CD45.1^+^Lin^−^ cells. In immune-suppression assays, co-culture with skin-derived CD45.1^+^ cells suppressed proliferation of CD3/CD28-stimulated T cells, reducing their carboxylfluorescein *N*-succinimidyl ester (CFSE)-dilution (33% on average). However, CD45.1^+^ cells isolated from BM allowed more than 86% of the co-cultured T cells to proliferate (Fig. 7b), to the same extent as did Lin^+^ cells isolated from inflamed skin (data not shown). Moreover, the suppressive activity of skin-derived CD45.1^+^ cells was relieved when an iNOS-inhibiting agent, NG-methyl-L-arginine acetate (L-NMMA)^31^, was added to co-cultures of activated T cells and skin-derived CD45.1^+^ cells (Fig. 7c). These results demonstrated that the Ly6G^int^Ly6C^+^ cells present in the inflamed skin would become MDSCs with an iNOS-dependent immune suppressive function^22^.

When the expression levels of anti- and pro-inflammatory genes were examined, it was revealed that both *iNos* and *Arginase-1*, representative genes related to MDSC function^22^, as well as the anti-inflammatory cytokine *Il10*, were expressed in skin-derived CD45.1^+^ cells at significantly higher levels, compared with their BM counterparts (Fig. 7d). Relatively higher expression of Arginase-1 and iNOS in the skin-derived CD45.1^+^ cells was confirmed by flow cytometric analysis (Fig. 7e). Also, higher expression of *Cd115* in the skin-derived CD45.1^+^ than the other cells (BM-counterparts and Lin^+^ cells) was verified. In the case of pro-inflammatory cytokines, *Tnfα* expression in the CD45.1^+^Lin^−^ progenies in inflamed skin was significantly lower than that in CD45.1^+^ Lin^+^ cells (Fig. 7d), while their *Il1β* expression was not significantly different from the *Il1β* expression in transplanted Lin^+^ cells. These results indicated the biased differentiation of transplanted Lin^−^ cells into MDSCs with immune-suppressive function in the inflamed skin.

Phenotypic profiles of host-derived CD45.1^−^CD11b^+^ cells on day 7 were very similar to those seen with transplanted Lin^+^ cells, with a predominant subpopulation of Ly6G^hi^Ly6C^low^, and low proportions of Arginase-1- or iNOS-positive cells within the host cells (Fig. S4, Supplementary). Numbers of host Ly6G^int^Ly6C^+^ and Ly6G^hi^Ly6C^low^ in inflamed skin were similar in the Lin^−^ recipients and in Lin^+^ recipients. Also CD115 expression profiles of the host CD11b^+^ cells in the Lin^−^ cell-recipients were similar to those in Lin^+^ cell recipients, both in the inflamed skin and BM. In addition, examination of skin-infiltrating cells of DNCB-sensitized mice revealed that inflammation induced myeloid differentiation of host stem cells has been initiated in response to the primary application of DNCB at the back skin. Thus, the difference in resolving rates of skin inflammation between Lin^−^ and Lin^+^ cell recipients could be attributed to Ly6G^int^Ly6G^+^CD115^+^ MDSC differentiation by the transplanted Lin^−^ cells than to host-driven myeloid/granulocytic cells.

Mesenchymal stem cells (MSCs) are also immune suppressive^32^ and comprised a proportion (≤10%) of Lin^−^ cells. However, we scarcely detected cells expressing MSC markers (CD34^−^CD45^−^CD105^+^Thy1.2^+^) among the skin-infiltrating cells of donor origin, when we transplanted Lin^−^ cells isolated from GFP-transgenic mice into dermatitis mice (Fig. S5, Supplementary). Moreover, when we transplanted Lin^−^ cells into dermatitis mice further depleted of CD105-expressing cells, we observed the same protective effect as that with transplantation of undepleted Lin^−^ cells. Thus, role of Lin^−^ cell differentiation into MSCs in this model is negligible compared with that for their differentiation into MDSCs.

Based on these data supporting the identification of Ly6G^int^Ly6C^+^ cells in the inflamed skin as MDSCs with immune-suppressive function, we examined the levels of T cell infiltration in the inflamed skin on day 7 post-transplantation of Lin^−^, Lin^+^ cells, or PBS, because pathogenesis of DNCB-induced dermatitis involves T-cell activation and skin infiltration^21^. Although CD3^+^ T cells were hardly detectable in flow cytometric analyses of skin-infiltrating cells on day 7 (data now shown), gene expression analyses of the inflamed skin tissues demonstrated that *Cd3* transcript levels in the inflamed skin were significantly lower in the mice receiving Lin^−^ cells than in those receiving Lin^+^ cells or PBS, indicating a relatively lower overall abundance of CD3^+^ T cells in the inflamed skin of the Lin^−^ cell-recipients. In support of this, flow cytometric analyses after staining the cells with anti-CD4 and -CD8 antibodies instead of anti-CD3 antibody demonstrated that proportions of CD4^+^/CD8^+^ cells in the skin-infiltrating cells were smaller in the Lin^−^ cell recipients than in the Lin^+^ cell or PBS recipients (Fig. 7g). This indicates the possibility of CD3-down-modulation by T cells activated in the inflamed skin. Variable levels of *Ly6g*, *Ly6c*, and *Cd115* were detected in the inflamed skin of each group, reflecting the relatively high frequencies of Ly6G^int^Ly6C^+^ CD115^+^ cells in the skin of Lin^−^ cell-recipients and of Ly6G^hi^Ly6C^low^ neutrophils in the skin of the Lin^+^ cell-recipients.

Together, these data demonstrate that differentiation of transplanted Lin^−^ cells in the inflamed skin was biased toward CD11b^+^Ly6G^int^Ly6C^+^CD115^+^ MDSCs with immunosuppressive functions in the inflamed skin, compared with the BM. Such skewed differentiation may be the cellular mechanism which promotes skin healing that follows Lin^−^ cell transplantation noted in recipient mice.

We then examined whether HSCs also suppress the skin inflammation upon transplantation, as did Lin^−^ cells. We depleted Lin^−^ cells of CD127^+^ and NK1.1^+^ cells to exclude any possible influence from innate like lymphoid (ILL) cells with immune suppressive function^33^,^34^ and isolated Sca-1-positive cells from these Lin^−^CD127^−^NK1.1^−^ cells to enrich the Sca-1^+^CD34^−/+^Lin^−^ long term/short term HSCs^35^ for transplantation into dermatitis mice (Fig. S6a, Supplementary). We found that transplanted Sca-1^+^Lin^−^CD127^−^NK1.1^−^ HSCs caused enhanced healing of the skin inflammation in the recipients, a little more effectively than Lin^−^ cells (Fig. S6b, Supplementary). In BLI analyses, these HSCs showed intensive expansion at the inflamed sites, just like Lin^−^ cells (Fig. S6c, Supplementary). CD11b/Ly6G/Ly6C profiles of the HSC progenies in the inflamed skin and BM were also similar to those of Lin^−^ cells on day 7 post-transplantation, with CD11b^+^Ly6G^int^Ly6C^+^ cells being the major population (Fig. S6d, Supplementary). We, therefore, concluded that effects on skin inflammation and *in vivo* dynamics of transplanted HSCs are similar to those of Lin^−^ cells.

## Discussion

In this study, via a longitudinal *in vivo* analysis of transplanted Lin^−^ cells in a murine model of contact dermatitis, we demonstrated that transplanted stem cells were recruited to the inflamed skin and underwent expansion and differentiation into CD11b^+^Ly6G^int^Ly6C^+^ immune-suppressive cells therein.

HSCs are recruited to sites of tissue damage in several injury and disease models^1^,^2^,^3^,^36^,^37^. Consistently, we showed recruitment of exogenous Lin^−^ cells to inflamed skin upon transplantation, as well as to BMs as major homing sites of stem cells. Through dynamics analyses, we further demonstrated that the recruitment of the exogenous Lin^−^ cells to the inflamed skin occurs directly, with maintenance of the Lin^−^ phenotype, following transplantation, without passing through the BM, although some portion of these cells also migrate to the inflamed tissue via the BM. Another intriguing point about the dynamics of infused Lin^−^ cells is that the progenies undergo *in situ* expansion in the inflamed skin, as confirmed by the increase in number of the progenies and the presence of BrdU-incorporating cells at the inflamed sites even following treatment with the BM-egress blockers FTY720 and DOP. Therefore, while most previous studies have focused on the recruitment and differentiation of HSCs at injured sites at fixed time points, our longitudinal study generates detailed information linking recruitment and subsequent *in situ* expansion and differentiation of exogenous HSCs.

Our systemic analyses of transplanted Lin^−^ cells in the dermatitis recipients revealed expansion of the progenies *in situ* in the inflamed skin and in the BM. Moreover, they undergo similar kinetics of myeloid differentiation at the two locations. These almost synchronous dynamics of transplanted Lin^−^ cells suggest that systemic soluble factors present in the recipients regulate such activities. Chemokines (e.g., CXCL12), growth factors (e.g., G/M-CSF), and pro-inflammatory cytokines (e.g., IFNα/β, TNF-α, and IL-1) produced at the site of inflammation induce mobilization of HSCs from the BM^3^,^5^. Pro-inflammatory cytokines such as IFN-α, IL-1 and IFN-γ could also induce HSCs and Lin^−^ cells to enter the cell cycle^38^,^39^,^40^. Moreover, these inflammatory cytokines promote biased differentiation of Lin^−^ cells into myeloid precursors and expansion of granulocytes^39^,^40^,^41^,^42^. Thus, we believe that the elevated levels of inflammatory cytokines, including TNF-α and IL-1, in the blood of DNCB-induced dermatitis mice (Fig. 1) regulate their recruitment to inflamed sites and subsequent expansion, as well as promote myeloid differentiation.

Importantly, we revealed that the majority of progenies of transplanted Lin^−^ cells were of the CD115^+^ CD11b^+^Ly6G^int^Ly6C^hi^ phenotype in the inflamed skin on days 5–7, when skin inflammation was most severe. Moreover, gene expression profiles and the suppressive function of these cells supported their identification as immune-suppressive MDSCs. In parallel flow cytometric analyses of CD45.2^+^ cells of recipient origin, the recipient-derived CD11b^+^ cells in inflamed skin had cellular compositions similar to those of the transplanted Lin^+^ cells (Fig. 6) on days 5 and 7 post-transplantation (data not shown). Therefore, reduced *Cd3* transcript levels in the inflamed skin and expedited skin regeneration in mice transplanted with Lin^−^ cells are concluded to be the outcomes of suppressive activity of the MDSCs newly generated from transplanted Lin^−^ cells, over T cell activation and inflammation. This suggested an immune-suppressive role of MDSCs in a skin inflammation model, consistent with previous reports of an anti-inflammatory function of CD11b^+^Ly6G^+^Ly6C^+^ MDSCs in disease models, including infection^43^,^44^.

The fact that differentiation of exogenous Lin^−^ cells into CD115^+^CD11b^+^Ly6G^int^Ly6C^hi^ cells was greater in the inflamed skin than in BM suggests that local inflammation enhanced this differentiation. For example, TLR/MyD88 signaling was related to MDSC expansion, as shown by significant expansion of suppressive MDSCs after high-dose LPS treatment in an airway disease model^45^. Consistently, when we transplanted Lin^−^ cells isolated from MyD88-deficient mice, the drastic cellular expansion in the inflamed skin usually observed after transplantation of wild-type Lin^−^ cells was impaired (unpublished data). Since homeostatic proteins, such as high mobility group box 1 and purinergic receptors, secreted into the extracellular milieu can induce TLR-mediated signaling^46^,^47^, local proteins released from the damaged skin tissues are considered to trigger *in situ* MDSC differentiation and expansion of Lin^−^ cells in the inflamed area.

In agreement with the view that MDSCs are at an immature differentiation stage^48^, CD11b^+^Ly6G^int^Ly6C^+^CD115^+^ MDSCs represented a major proportion of Lin^−^ progeny on days 5 and 7, when these progenies showed extensive proliferation *in situ* and before the emergence of other cell lineages on day 14, highlighting the extreme plasticity and proliferative potential of the MDSCs. On day 14 post-transplantation, Ly6G levels in the CD11b^+^Ly6G^int^Ly6C^+^ cells were slightly increased, and CD11b^+^Ly6G^hi^Ly6C^int^ neutrophils became the predominant population. As neutrophils have a very short life span^49^, the decrease in luminescence signals at the site of inflammation following the peak at day 14 post-transplantation can be ascribed to differentiation of B6.LucTg Lin^−^ cells into short-lived cell types, such as neutrophils. However, faint signals remained evident on days 35 and 42 post-transplantation (data not shown), although luminescence signals were significantly diminished at both inflamed sites and in BM by day 28 post-transplantation (Fig. 2c). These results suggest that certain rare progenies of transplanted Lin^−^ cells may persist in the regenerated skin and BM as stem cells. Alternatively, the cells emitting the faint signals may be those that have trans-differentiated into parenchymal cells incorporated into the dermis, producing collagen III^15^ and/or other cellular components necessary for skin regeneration^50^. Further studies are necessary to characterize the cells emitting the reminiscent signals in the inflamed skin after the inflammation subsided significantly and to provide direct evidence of trans-differentiation in the context of dermatitis mice. Nonetheless, this study clearly demonstrates that the therapeutic effects and enhanced skin regeneration facilitated by Lin^−^ cells stems from their *in situ* differentiation into anti-inflammatory MDSCs.

Collectively, we demonstrate dynamic migration, expansion, and differentiation of transplanted Lin^−^ cells in recipients with skin inflammation, suggesting that skewed differentiation into immune-suppressive CD11b^+^Ly6G^int^Ly6C^+^MDSCs with enhanced CD115 expression correlates the therapeutic effect of transplanted Lin^−^ cells. This study provides an overview of the *in vivo* dynamics and insights into the activity of transplanted HSCs, and suggests that HSC transfer can be a potential treatment modality for serious diseases such as psoriasis and in skin graft-versus host disease (GVHD).

## Methods

### Mice

C57BL/6 (B6), B6.SJL-*Ptprc*^*a*^*Pep3*^*b*^/BoyJ (CD45.1^+^), B6(Cg)-*Tyr*^c−2J^/J (tyrosinase mutant B6 mice; B6.Albino), and C57BL/6-Tg (CAG-EGFP)10sb/J (GFP- transgenic) mice were purchased from the Jackson Laboratory (Bar Harbor, ME, USA). The transgenic luciferase mouse line (B6-Tg[CAG-effLuc]; B6.Luc-Tg), which expresses a modified firefly luciferase gene under the control of the actin promoter, has been described previously^18^ and was maintained by crossing with B6 mice. All experiments were performed using mice between the ages of 8 and 12 weeks, approved by the Institutional Animal Care and Use Committee of Seoul National University, and carried out in accordance with the approved guide line. Mice were maintained under specific pathogen-free conditions at the Center for Animal Resource Development of Seoul National University, College of Medicine, Korea.

### Induction of skin dermatitis

Mice were sensitized on day 0 by epicutaneous application of 100 μL 2% DNCB (Sigma-Aldrich, St. Louis, MO, USA) in a 4:1 acetone:olive oil solution onto dry shaved back skin, as described previously^5^; a 4:1 combination of acetone and olive oil was used as the vehicle control. After 5 days, an identical volume of 2% DNCB solution was applied to both sides of the right ear of the test group (n = 5), while the control group (n = 5) received the vehicle (acetone/olive oil). Clinical severity scores were graded on a scale of 0 to 4 regarding four criteria, erythema, scarring, edema, and excoriation with grading as follows: erythema (0, no sign; 1, almost clear with slight redness; 2, mild erythema with definite redness; 3, moderate erythema with marked redness; 4, severe erythema with fiery redness), scarring (0 described as follows. 0, no sign; 1, no change in ear shape, but with a little scar observed; 2, a little change in ear shape, with a little crooked and moderate scar; 3, loss of ear marginal area with very severe scars; 4, loss of whole ear shape), edema (ear thickness is below 0.1 mm in 0; 1, 0.1–0.2 mm; 2, 0.2–0.3 mm; 3, 0.3–0.4 mm; 4, >0.4 mm), and excoriation (0, no signs; 1, scaling observed; 2, marginal epidermal loss; 3, epidermal loss; 4 epidermal and dermal layer loss with bleeding). All the grades were added up for clinical scores.

### Histological analysis

Skin specimens were obtained on day 28 and fixed in 4% formaldehyde. Tissues were cut mid-sagittally, embedded in paraffin 24 h after fixation, and serially sectioned for histological analysis. Tissue sections were stained with hematoxylin and eosin (H&E) for analysis of general morphology.

### Cell preparation

Bone marrow cells were isolated from the femurs and tibiae of 8-week-old B6 mice. BM Lin^−^ cells were purified by magnetic cell sorting (MACS) according to the manufacturer’s instructions (Miltenyi Biotec, Auburn, CA, USA), using a BD Pharmingen Biotin Mouse Lineage Panel (San Jose, CA, USA). Isolated Lin^−^ cells (2 × 10^6^), with or without CMFDA (5 μM; Invitrogen, Carslad, CA, USA)-labelling, were i.v. injected to dermatitis mice one day after ear challenge with DNCB. For depletion of CD105^+^ cells, purified Lin^−^ cells were stained with an APC-conjugated anti-CD105 antibody (clone MJ7/18; eBioscience, San Diego, CA, USA) and then magnetically labeled using anti-APC microbeads (Miltenyi Biotec), followed by negative selection using the MACS system. The negative fractions were harvested, washed and adjusted to the appropriate cell numbers for injection in PBS. For isolation of Sca-1^+^Lin^−^CD127^−^NK1.1^−^ cells, purified Lin^−^ cells were negatively selected using MACS after staining with PE-conjugated anti-CD127 (A7R34; eBioscience) and anti-NK1.1(PK136; eBioscience) and magnetic labeling with anti-PE microbeads, and then positively selected after staining with APC-conjugated anti-Ly6A(Sca-1; D7; eBioscience) and labeling with anti-APC microbeads. The positive fractions were harvested, washed and adjusted to the appropriate cell numbers for injection in PBS. Each purified cells (0.5 – 5 × 10^6^) were i.v. injected with in 200 μl of PBS. Leukocytes infiltrating skin were isolated as described previously^48^. In brief, skin (1 × 1 cm^2^) was cut into small pieces, digested with 1 mg/ml collagenase type II (Sigma-Aldrich) and 100 mg/ml DNase (Sigma-Aldrich) for 60 minutes at 37 °C, and then re-suspended in staining buffer (1 × PBS with 0.1% bovine serum albumin and 0.1% sodium azide) after terminating digestion by adding staining buffer and centrifugation. For FACS sorting of transplanted CD45.1^+^ cells, freshly isolated cells were incubated with anti-mouse CD45.1-PE (eBioscience) for 20 min at 4 °C. Cells were washed with FACS buffer and sorted on a FACS Aria flow cytometer (BD Pharmingen).

### Bioluminescence imaging (BLI) analysis

Tyrosinase mutant B6 mice (B6.Albino) were used as a model for skin dermatitis BLI analysis, because their white coat color facilitated detection, without absorption, of luminescence signals. *In vivo* bioluminescence imaging was performed using an IVIS 100 imaging system with a charge-coupled device (CCD) camera (Caliper Life Science, Waltham, MA, USA), as described previously^19^. Mice on the imaging stage were given an i.p. injection of the substrate, D-luciferin (150 mg/kg body weight; Molecular Probes, Eugene, Oregon, USA) under anesthesia using 1.5% isoflurane gas in oxygen at a flow rate of 1. L/min. Relative intensities of emitted light were presented as pseudocolor images ranging from red (most intense) to blue (least intense), which were superimposed on gray-scale photographs using the Living Image (ver. 2.12; Xenogen, Alameda, CA, USA) and IGOR (WaveMetrics, Portland, OR, USA) image analysis software packages. Signal intensities emitted by ROI were measured and expressed as photon fluxes (photon s^−1^cm^−1^sr^−1^), which refer to the photons emitted from a solid angle of a sphere. Data are presented as means ±standard error of the mean (SEM). Instrument background was subtracted electronically, both from the images and from the measurements of photon flux.

### Flow cytometric analysis

Single-cell suspensions of BM and skin-infiltrating leukocytes were incubated at 4 °C for 20 min in staining buffer (1 × PBS with 0.1% bovine serum albumin and 0.1% sodium azide) containing the appropriate antibody mix. NOS2-AF488, CD45.1-eFluor 450, Ly6G-FITC, Ly6G-PE, Ly6G-PE.Cy7, CD11b-PE, CD11b-APC, F4/80-PE, CD115-APC, Ly6C-APC, CD45-PE, B220-PE, CD4-APC, CD8-APC, and CD105-APC antibodies were obtained from eBioscience. Lineage cocktail-APC, CD11c-APC.Cy7, α-BrdU-FITC and CD34-PE antibodies were purchased from BD Pharmingen (San Diego, CA, USA). Arginase-FITC antibody was purchased from R&D systems (Minneapolis, MN, USA). For the flow cytometric analysis of Lineage-positive cells, the cells were stained with biotinylated Lineage cocktail obtained from BD Pharmingen, then labeled with streptavidin-V450 (BD Pharmingen). Data were collected using a FACSCalibur or LSRII-Green (both BD Pharmingen) and were analyzed using the FlowJo software (Tree Star, Ashland, OR, USA).

### Administration of drugs and antibodies

Mice were treated with FTY720 (2 μg/mL; Sigma-Aldrich) or 4-deoxypyrodixine hydrochloride (DOP; 30 μg/mL; Sigma-Aldrich) by direct addition to their drinking water. For the BrdU-incorporation assay, mice were i.p. injected with 1 mg BrdU (Sigma-Aldrich) once daily for 4 days. For depletion of Gr-1-expressing cells, mice were injected i.p. with 0.1 mg of either a rat anti-mouse Gr-1 antibody (BioXcell, Lebanon, NH, USA) or a control rat anti-mouse IgG antibody (BioXcell).

### Quantitative RT PCR (qRT-PCR)

RNA from the transplanted CD45.1^+^ cells or from total cells infiltrating inflamed skin or in BM, or whole tissues of the inflamed right ear were isolated using the RNeasy Micro RNA Kit (Qiagen, Valencia, CA, USA) and reverse-transcribed to generate cDNA using M-MLV reverse transcriptase (Takara Bio, Otsu, Shiga, Japan), 1 × RT buffer, 0.25 mM dNTP, and 40 units RNase inhibitor (Koschem Co., Seoul, Korea). Quantitative PCR using the cDNA as a template was performed as described by the manufacturer of SYBR premix ExTaq (Takara Bio) in 96-well plates on a thermal cycler (Light Cycler 96; Roche, Mannheim, Germany). Relative expression levels were calculated using the 2^−ΔΔCt^ method after normalization to β-actin. The primer sequences are as follows: *Arg1*, forward 5′-ctccaagccaaagtccttagag-3′, reverse 5′-aggagctgtcattagggacatc-3′; *Nos2*, forward 5′-gttctcagcccaacaatacaaga-3′, reverse 5′-aggagctgtcattagggacatc-3′; *Il10*, forward 5′-cagagaagcatggcccagaa-3′, reverse 5′-gtcaaattcattcatggccttgt-3′; *Tnfα*, forward 5′-ggaacacgtcgtgggataatg-3′, reverse 5′-ggcagactttggatgcttcttt-3′; *IL1β*, forward 5′-gcaactgttcctgaactcaac-3′, reverse 5′-atcttttggggtccgtcaact-3′; *Cd3*, forward 5′-atgcggtggaacactttctgg-3′, reverse 5′-gcacgtcaactctacactggt-3′; *Ly6g*, forward 5′-gtcccccaagccttgtgtg-3′, reverse 5′-aggtacttgtttagtgggaggg-3′; *Ly6c*, forward 5′-gcagtgctacgagtgctatgg-3′ , reverse 5′-actgacgggtctttagtttcctt-3′; *Cd115*, forward 5′-tgtcatcgagcctagtgg-3′, reverse 5′-cgggagattcagggtccaag-3′; *b-actin,* forward 5′-ggctgtattcccctccatcg-3′; reverse, 5′-ccagttggtaacaatgccatgt-3′.

### ELISA

Serum concentrations of TNF-α and IL-1β were measured by ELISA, according to the manufacturer’s protocol (BD Biosciences) and read at 450 nm using a microplate reader (BioTek, Winooski, VT, USA). All samples and standards were run in triplicate.

### Immune suppression assays

Immune suppression was evaluated by CFSE-dilution. Splenocytes from DNCB-treated mice were labeled with CFSE (2.5 μM; Invitrogen), stimulated with anti-CD3 (2 μg/ml; eBioscience)/CD28 (1 μg/ml; eBioscience) antibodies, and co-cultured at a 1:1 ratio with CD45.1^+^ cells sorted from inflamed skin or BM of dermatitis mice transplanted with CD45.1^+^ Lin^−^ cells for 3 days. For inhibition of iNOS activity, L-NMMA (1 mM, Sigma-Aldrich) was added at the beginning of co-culture. Then, the cells in co-culture were stained for surface marker expression with CD3-APC (eBioscience) and analyzed for CFSE-dilution by the CD3-positive cells in flow cytometry.

### Statistical analysis

The statistical analyses were performed using GraphPad Prism ver. 5, (GraphPad Software, San Diego, CA, USA). *P* values were determined using two-tailed unpaired Student’s *t*-tests; *P < 0.05, **P < 0.01, ***P < 0.001). Data are presented as means ±standard error of the mean (SEM).

## Additional Information

**How to cite this article**: Jeong Ryu, S. *et al.* Alleviation of skin inflammation after Lin^–^ cell transplantation correlates with their differentiation into myeloid-derived suppressor cells. *Sci. Rep.* **5**, 14663; doi: 10.1038/srep14663 (2015).

## Supplementary Material

Supplementary Information
