# Supplementary material for: Alleviation of skin inflammation after Lin− cell transplantation correlates with their differentiation into myeloid-derived suppressor cells
Source: Sci Rep. 2015 Oct 6;5:14663. doi: 10.1038/srep14663 (PMC4594128; doi:10.1038/srep14663)
Supplement: Supplementary Information [file srep14663-s1.pdf]

Alleviation of skin inflammation after Lin$^-$ cell transplantation correlates with their differentiation into myeloid-derived suppressor cells

Su Jeong Ryu[1], Ji-Min Ju[1], Woojin Kim[1], Min Bom Kim[1], Kuen Hee Oh[1], Dong Sup Lee[1], Hakmo Lee[2], Ju Eun Oh[3] Kyong Soo Park[4], Eun Young Choi[1*]

1. Department of Biomedical Sciences, Seoul National University College of Medicine, Seoul, Korea

2. Biomedical Research Institute, Seoul National University Hospital, Seoul, Korea.

3. Department of Molecular Medicine and Biopharmaceutical Sciences, College of Medicine or College of Pharmacy, Seoul National University, Seoul, Korea

4. Department of Internal Medicine, Seoul National University College of Medicine, Seoul, Republic of Korea.

Supplementary Figure S1

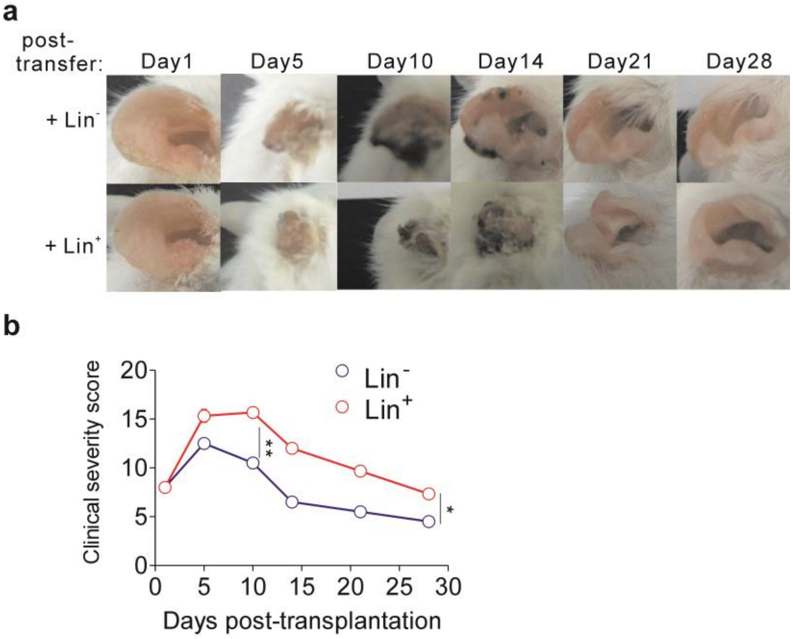

Supplementary Figure S1. Transplantation of Luc-Tg Lin$^-$ cells enhances healing and skin regeneration in dermatitis mice

(a) Lin$^-$ or Lin$^+$ cells isolated from the BM of Luc-Tg mice were injected (5×10^5) i.v. into B6-Albino mice treated with DNCB. Longitudinal photos of challenge ear of same mouse are presented. (b) Longitudinal clinical scores of these mice are presented. Data (a-b) represent more than three independent experiments (n = 4 mice/group/experiment). Data (b) are presented as means ± SEM. *P* values were determined using two-tailed unpaired Student's *t*-tests; $^*P < 0.05$, $^{**}P < 0.01$, $^{***}P < 0.001$.

Supplementary Figure S2

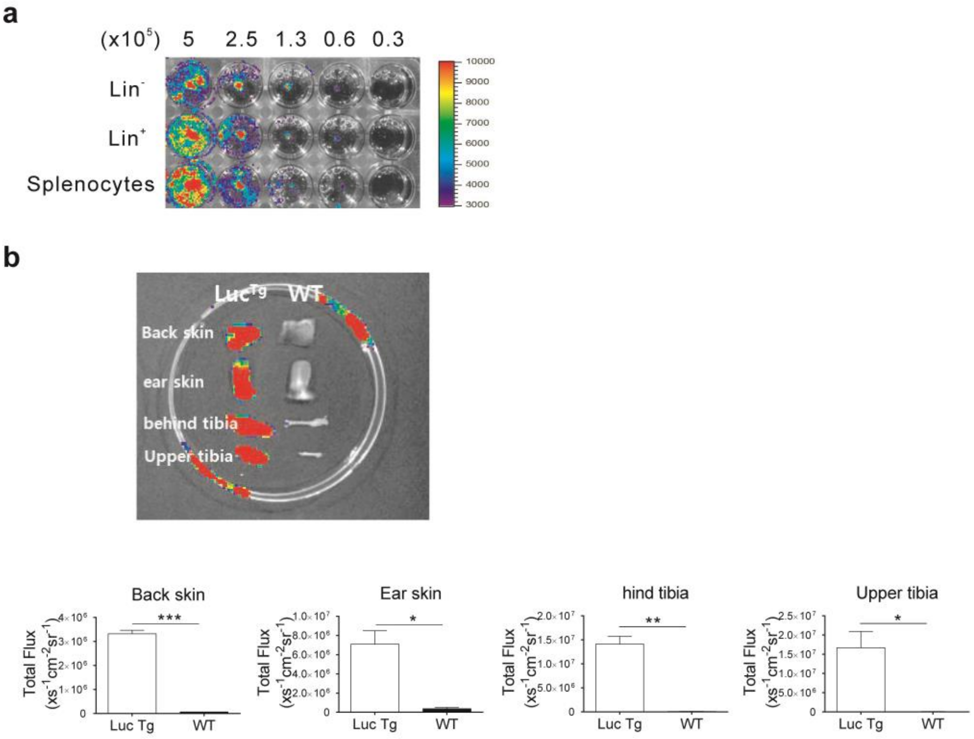

Supplementary Figure S2. Luminescence assays of cells and tissues from Luc-Tg mice. (a) Luminescence assay using Lin^- and Lin^+ cells, and splenocytes isolated from Luc-Tg mice. Different numbers of single cell suspensions were plate onto wells of 24-well plates. Luminescence signals were measured 10 min after luciferin addition to the wells. (b). Luminescence images of different tissues from Luc-Tg mice. Luc-Tg or WT mice were injected i.p. with luciferin and were perfused 5 min later. Back and ear skin, and upper and hind leg tibia were prepared from the perfused mice and taken for images. Data (a-b) represent more than three independent experiments (n = 3 mice/group/experiment). The photon values are presented as means ± SEM. *P* values were determined using two-tailed unpaired Student's *t*-tests; *$P < 0.05$, **$P < 0.01$, ***$P < 0.001$.

Supplementary Figure S3

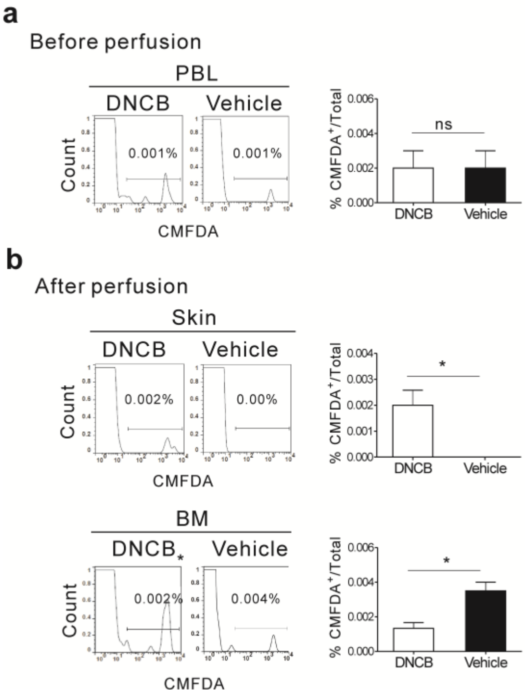

Supplementary Figure S3. Detection of CMFDA$^+$ Lin$^-$ cells at 12 hr after transplantation.

(a) Analysis of PBLs from the dermatitis or control mice transplanted with CMFDA$^+$ Lin$^-$ cells. PBLs were prepared from the transplanted mice at 12 hr post-transplantation to detect CFMDA$^+$ cells in blood by flow cytometric analysis. (b) Analysis of BM and skin-infiltrating cells after mouse perfusion. After bloods were taken for PBL analysis (a), transplanted mice were perfused with PBS and, then, were prepared for analysis of skin- and BM-infiltrating cells via flow cytometry. Representative flow cytometric data (a-b) from two independent experiment (n=3/group/experiment) are shown. Percentage values of CMFDA$^+$ cells in the infiltrating are plotted. Data (a,b) are presented as means ± SEM. *P* values were determined using two-tailed unpaired Student's *t*-tests; **P* < 0.05, ***P* < 0.01, ****P* < 0.001.

Supplementary Figure S4

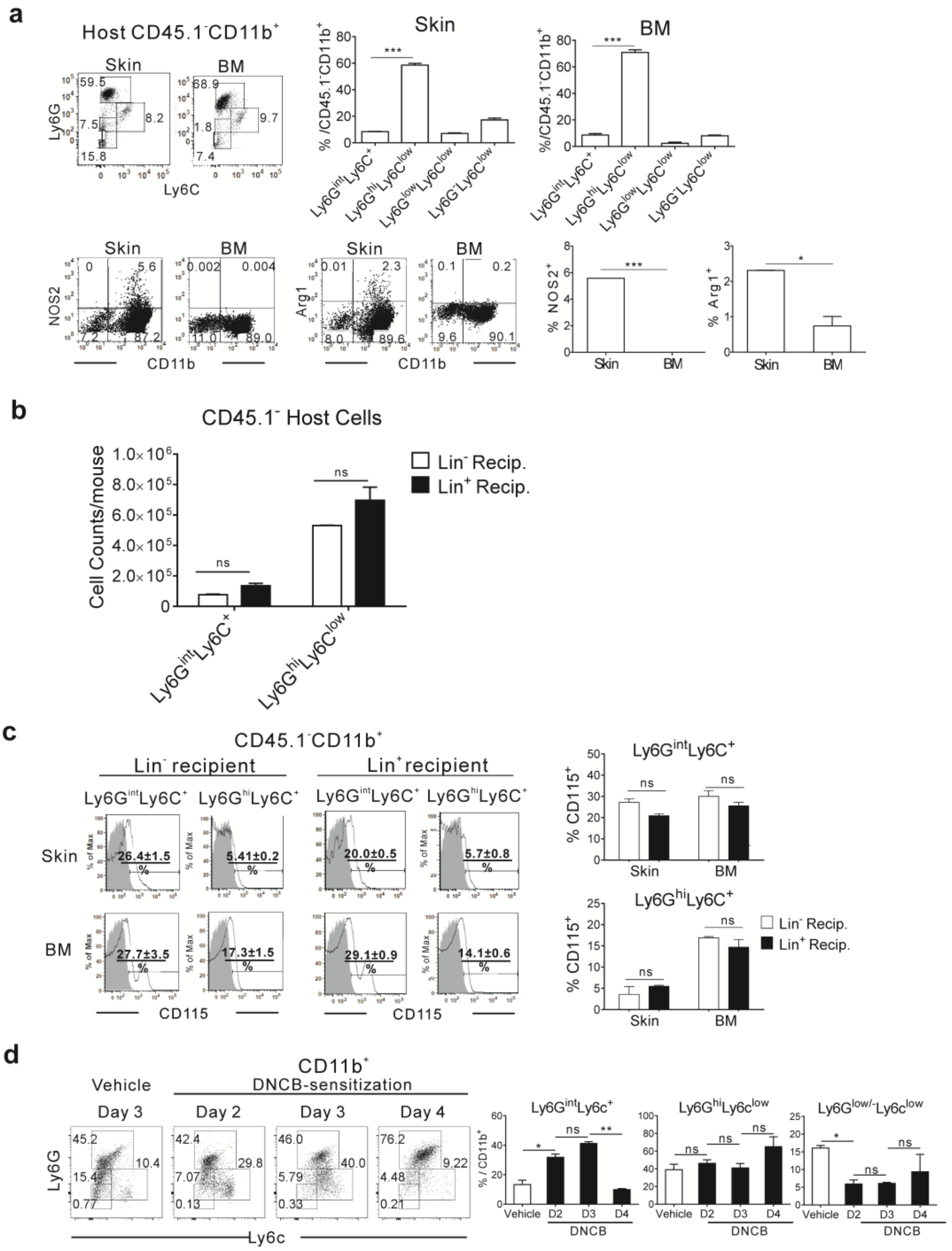

Supplementary Figure S4. Host CD45.1$^-$CD11b$^+$ cells in the dermatitis mice with transplantation of Lin$^-$ or Lin$^+$ cells.

(a) Phenotypes of host CD45.1$^-$CD11b$^+$ cells in inflamed skin. Flow cytometric analysis was done for Ly6G/Ly6C profiles of CD45.1$^-$CD11b+ cells in the inflamed skin and their expression of iNOS and Arginase on day 7 post-transplantation of Lin$^-$ cells. Representative data are shown. Proportions of each subpopulation and cells expressing iNOS and Arginase-1 are plotted. (b) Numbers of Ly6G^{int}Ly6C$^+$ and Ly6G^{hi}Ly6C^{low} cells of host origin in the inflamed skin of Lin$^-$ or Lin$^+$ cell recipients on day 7 post-transplantation. (c) Expression of CD115 by host CD45.1$^-$CD11b$^+$ cells. CD45.1$^-$CD11b$^+$ cells from inflamed skin and BM of Lin$^-$ and Lin$^+$ cells recipients were analyzed for their expression of CD115. Representative data are shown. Percentages of CD115$^+$ cells in Ly6G^{int}Ly6C$^+$ and Ly6G^{hi}Ly6C^{low} cells are plotted. (d) Phenotypic analysis of host CD11b$^+$ cells present in the sensitized skin after primary DNCB-sensitization. Skin-infiltrating cells were harvested on days 2, 3, and 4 after DNCB-application at the back skin, and analyzed for their Ly6G/Ly6C profiles. Representative flow cytometric data are shown. Data from control mice with vehicle treatment are shown in parallel. Proportions of different subpopulations are plotted. Data (a-d) are representative of two independent experiments (n = 3-5 mice/group/experiment). Data (a-d) are presented as means ± SEM. *P* values were determined using two-tailed unpaired Student's *t*-tests; *$P < 0.05$, **$P < 0.01$, ***$P < 0.001$

Supplementary Figure S5

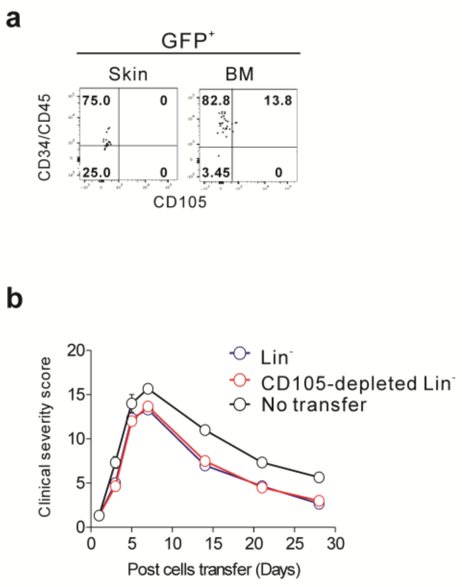

Supplementary Figure S5. Examination of the influence of CD105^{+} MSC on the healing of skin inflammation after Lin^{-} cell transplantation. (a) Lack of CD105-expressing cells in the inflamed skin of dermatitis mice transplanted with Lin^{-} cells. Skin-infiltrating cells or BM cells in dermatitis recipients were with analyzed for the presence of CD34^{-}CD45^{-}CD105^{+} cells of donor origin (GFP-positive) via flow cytometry on day 7 post-transplantation of Lin^{-} cells isolated from GFP-transgenic mice. Data shown are gated on GFP-positive cells and represent two independent experiments (n = 5 mice/experiment). (b) Disease scores of the dermatitis mice transplanted with the Lin^{-} cells or CD105-depleted Lin^{-} cells. Dermatitis mice were transplanted with Lin^{-} cells depleted of CD105-expressing cells, undepleted Lin^{-} cells, or PBS (no transfer) according to the schedule described in Fig. 1. Skin inflammation scores of the three groups of mice were graded periodically and were plotted longitudinally. The graph represents two independent experiments (n = 5/group/experiment).

Supplementary Figure S6

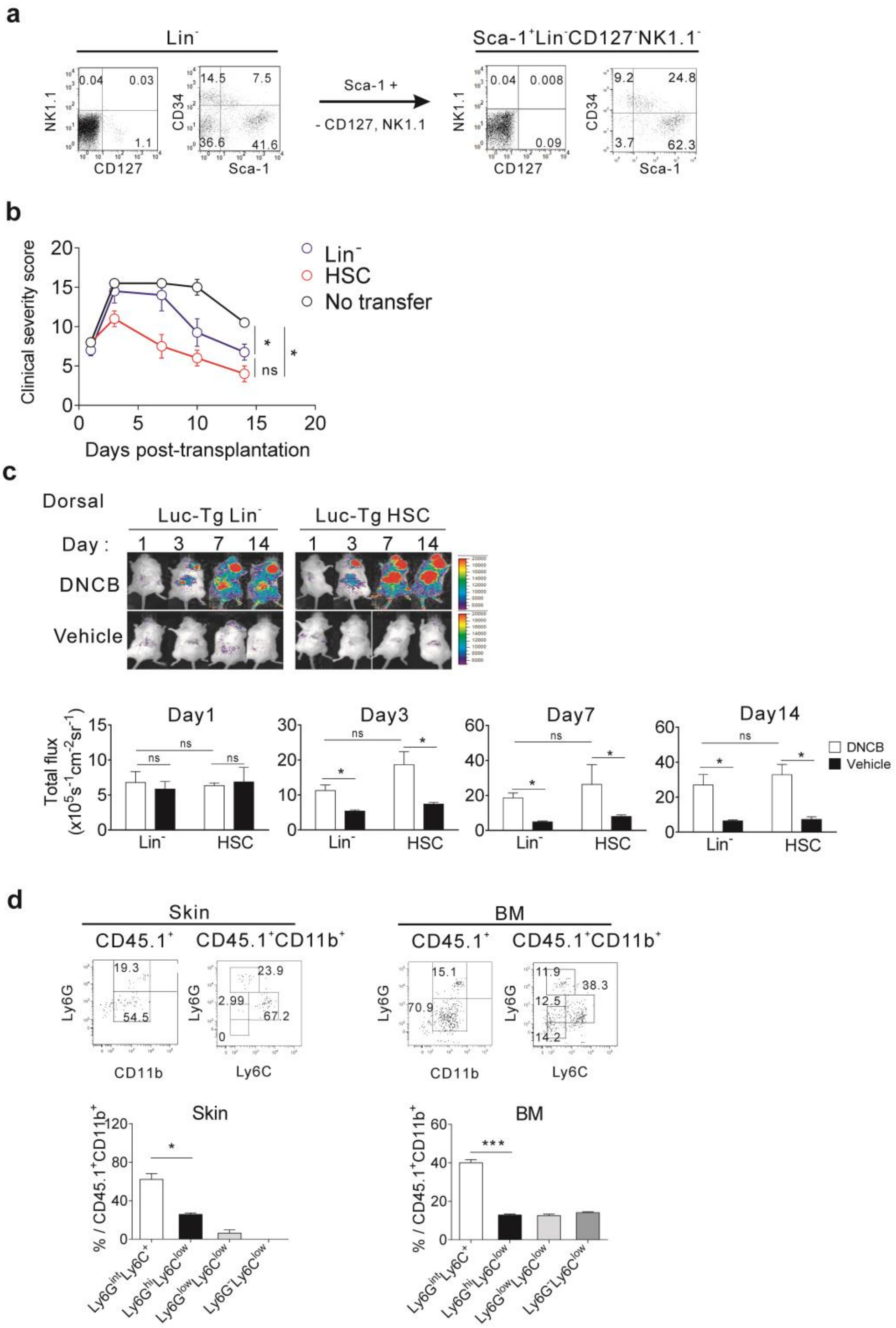

Supplementary Figure S6. Therapeutic effects and in vivo dynamics of HSCs transplanted to dermatitis mice.

(a) Purification of Sca-1^{+}Lin^{-}CD127^{-}NK1.1^{-} HSCs from CD45.1^{+} mice. Lin^{-} cells from BM of CD45.1^{+} mice were further depleted of CD127^{+}NK1.1^{+} cells and Sca-1-positive cells were isolated from the Lin^{-}CD127^{-}NK1.1^{-} cells. Lin^{-} cells before (left) and after (right) the further procedures were analyzed by flow cytometry and representative data are shown. (b) Longitudinal clinical scores after transplantation. Sca-1^{+}Lin^{-}CD127-NK1.1^{-} HSCs, Lin^{-} cells, or PBS were i. v. injected (1×10^{6}) to DNCB-treated mice and the mice were periodically checked (n = 5 mice/group/experiment). (c) BLI analysis of HSCs (Sca-1^{+}Lin^{-}CD127-NK1.1^{-}) and Lin^{-} cells in the DNCB-treated mice (n = 4 mice/group/experiment). These cells (2.5×10^{5}) were purified from Luc-Tg mice and were transplanted into dermatitis mice. Dorsal images of the recipients are shown. The photon values of ear skin as ROI are plotted. (d) Flow cytometric analyses of HSCs progenies in inflamed skin and BM of the dermatitis mice (n = 3 mice/group/experiment). On day 7 after transplantation of 1×10^{6} HSCs (Sca-1^{+}Lin^{-}CD127^{-}NK1.1^{-}) purified from CD45.1^{+} mice, CD45.1^{+} cells infiltrating the inflamed skin and BM of the recipients were prepared and analyzed by flow cytometry. Representative flow cytometric data are presented, and the percentages of each subpopulation are plotted. Data shown (a-d) represent two independent experiments. Data (b-d) are presented as means ± SEM. *P* values were determined using two-tailed unpaired Student's *t*-tests; $*P < 0.05$, $**P < 0.01$, $***P < 0.001$.
